# Bilateral Cochlear Implantation in Meningitis, Is it Mandatory? A Case Report

**Published:** 2012-08-30

**Authors:** Mohammad Ajalloueyan, Susan Amirsalari, Shahla Afsharpeyman

**Affiliations:** 1 Research Centre for New Hearing Technologies, Baqiyatallah University of Medical Sciences, Tehran, IR Iran

**Keywords:** Cochlear Implants, Meningitis

## Abstract

These days cochlear implantation is the accepted modality to rehabilitate deafened people. Meningitis is still a life threatening disease which may lead to deafness due to sole disease or secondary to ototoxic drugs used to stop the disaster [1]. Sepsis and/or meningitis may harm neonates whom are taking care in nurseries. TEOAE neonatal hearing screening programs are unable to find all of these deafened children and ABR would be necessary to explore most of them [2].Cochlear implantation should be performed as soon as possible and before complete ossification of cochlea.

## 1. Introduction

The cornerstone to rehabilitate deaf victims is cochlear implantation. Time to perform bilateral implantation is a challenging issue. Cochlear obliterance after bacterial meningitis enhances electrode insertion and efficiency. This case report confirms the idea of bilateral cochlear implantation as early as possible.

## 2. Case Report

**Figure 1:**
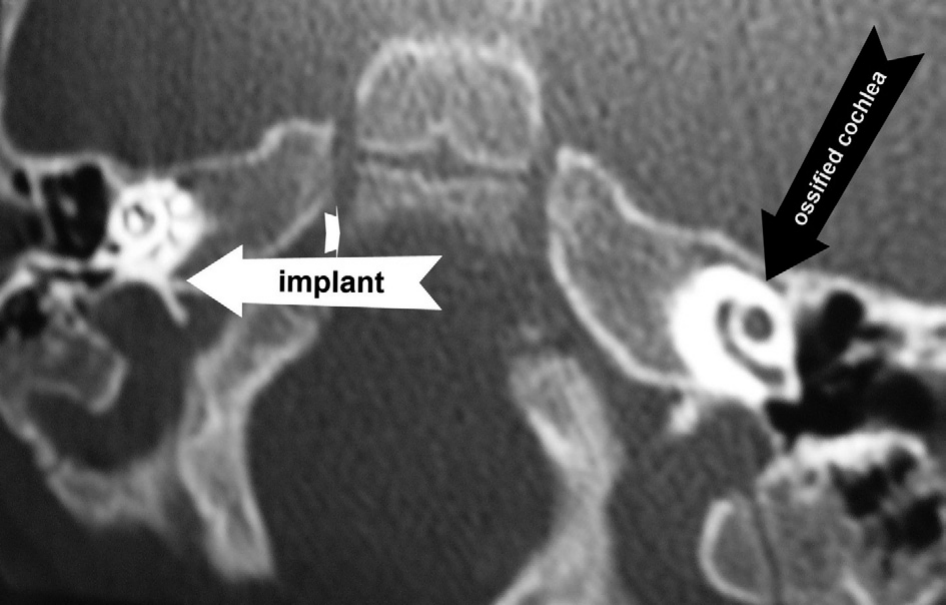
Nucleus® contour electrode in R side and cochlear ossification in L side

In Sep 2010 a 4 month girl was brought to our cochlear implant centre after discharging from another hospital with deafness after proven pneumococcal meningitis. She took Imipenem® and Vancomycin® during hospital course without harsh on kidney, liver or blood. There was not any familial history of deafness or proven history of TORCH infection. TEOAE after birth was passed but new TEOAE after meningitis was failed and ABR could not detect any wave at 90 dB Hearing Level. Second ABR two months later was same as before. Her family accepted the suggestion for cochlear implantation and she was taken to operating room just three months after meningitis recovery. Unfortunately family found for CI was restricted to do bilateral implantation at that time. One year later when family could prepare for second ear surgery new CT scan revealed ossification and near complete obliteration of cochlea (Figure 1).Now her unilateral hearing is acceptable and speech rehabilitation is going on.

## 3. Discussion

Although our patient’s general health condition and hearing and speech rehabilitation is favorable, she might have lost the chance to have bilateral hearing [3]. Some data indicate that cochlear ossification after pneumococcal meningitis starts as soon as three weeks after meningitis [4] and others believe that bilateral CI should not be postponed for more than three months [5][6]. Delay in performing CI may impede electrode insertion due to cochlear ossification and negatively impact speech and communication outcome [7]. The option to place a dummy electrode to keep a space in the cochlea may also be acceptable. Was a MRI performed at the time of the first implant might have shown fluid in the cochlea or possible ossification. Usually ossification is nearly complete by 3 months (the time at which the first implant was performed). Our experience in this case confirms the above findings too, so we recommend early bilateral CI in post meningitis deafened children.
